# Pregnancy Outcomes in Autoimmune Rheumatic Diseases: A Persistent Clinical Challenge

**DOI:** 10.31138/mjr.031125.apr

**Published:** 2025-12-31

**Authors:** Styliani Partalidou

**Affiliations:** Fourth Department of Internal Medicine, Hippokration General Hospital, School of Medicine, Aristotle University of Thessaloniki, Thessaloniki, Greece

**Keywords:** connective tissue disease, pregnancy, adverse outcomes, rheumatoid arthritis, systemic sclerosis

The majority of autoimmune rheumatic diseases are more common in women of child-bearing age; thus, their effect in sexual functionality, fertility, pregnancy course, and outcomes, as well as the impact of the pregnancy on disease activity, is of great importance. Such patients are advised to conceive during remission, or at least low disease activity in order to avoid adverse events and disease relapse. For these reasons, preconception counselling and family planning is necessary. Even so, these pregnancies are still considered high-risk and need close monitoring by a multidisciplinary team, including obstetricians, rheumatologists, neonatologists and midwives, both throughout the pregnancy and postpartum period.^1^ Although underestimated, sexual dysfunction is a usual problem in patients with rheumatic diseases. Pain and depression are the leading causes.^2,3^ These factors should also be taken into account, as they would constitute avoidant factors for reproduction in this population.

The therapeutical agents used in women with rheumatic diseases desiring to become mothers, should be carefully considered in advance, as limitations with pregnancy compatibility exist for some of the drugs used in daily clinical practice, including biological disease modifying anti-rheumatic drugs (bDMARDs) and oral small molecules, such as Janus Kinase (JAK) inhibitors.^4^ Furthermore, methotrexate, the anchor drug in the management of rheumatoid arthritis (RA) should be discontinued three to six months before conception, making family planning, again, urgent.^5^ Breastfeeding is generally encouraged, given that the status of mother postpartum is good and compatible medications are prescribed.^6^

Clinical experience and data from previous studies suggest that RA tends to improve or remain stable during pregnancy course.^7^ Patients with seronegative autoantibody status usually experience the greatest improvement.^8^ Considering pregnancy outcomes, literature implies that caesarean section (C-section), hypertension, and intrauterine growing restriction (IUGR) are increased compared with the general population.^9^ Postpartum relapse is usual, possibly due to the abrupt decrease of circulating endogenous cortisol levels.^7^ Systemic sclerosis (SSc), also, tends to remain stable, although data are scarce in contrast to RA. Some clinical manifestations, such as Raynaud phenomenon, however, improve due to the generalized vasodilation. The pregnancy outcomes are again adverse, with miscarriage, low-birth weight neonates and pre-term delivery being the most common.^10^ The rare case of pre-eclampsia is highly difficult to differentiate from scleroderma renal crisis, requiring renal biopsy to establish a definite diagnosis.^11^ Whether SSc follows the same pattern with RA, with exacerbation after labour, remains unclear, mainly due to limited data and the fact that patients are often of more advanced age or even in menopause during disease progress.

The above-mentioned findings are the result of specific cellular changes during pregnancy. T-regulatory cells expand to minimize foetal abortion and T-cells shift from Th1 to Th2 phenotype, which, in turn, results in a shift to the expressed interleukins (IL). More specifically, tumour necrosis factor a (TNF-a), interferon-γ (IFN-γ) and IL-12 are decreased, while IL-4, IL-5 and tumour-growth factor β (TGF-β) are increased. These cellular modulations explain why Th1-mediated diseases, such as RA present a more favourable course, while Th2-mediated conditions, like Systemic Lupus Erythematosus (SLE) tend to exacerbate.^12,13^

In the current issue of Mediterranean Journal of Rheumatology (MJR), there are two interesting prospective studies from India and Qatar, highlighting how pregnancies in women suffering from SSc and RA respectively, yield a high risk of adverse pregnancy outcomes, namely, miscarriage, IUGR neonates, pre-eclampsia, pre-term delivery, and C-section.^14–15^

In the Al Emadi et al. study, the percentage of women with active disease (CDAI>2.8) decreased towards third trimester, indicating disease improvement, as expected based on previous studies. However, women still experienced unfavourable outcomes, with pre-term delivery being the anchor event, followed by miscarriage and C-section.^14^ The findings of this study get along and confirm the existing literature. It is noteworthy, though, that gestational diabetes was significantly more prominent in the control group, despite the use of corticosteroids in RA patients. The use of hydroxychloroquine may be the answer to this phenomenon, as it is known for its beneficiary effects on cardiometabolic components, and the researchers report that it was the most usually prescribed agent.

In the Indian SSc cohort, the pregnancy outcomes were similarly adverse, with high pregnancy loss, compared to the general population, although the comparison of outcomes between pre-disease and post-disease pregnancies did not differ.^15^ This finding is consistent with the results of other studies, reporting that SSc patients both prior and after SSc diagnosis are of increased risk for spontaneous abortion compared with healthy controls.^16^ This finding may underline the preclinical immunological dysregulation contributing to increased adverse events.

These two studies, published in the present issue of MJR, are in agreement with previously published works, contributing to more confident reassurance of the results, as the number of patients were limited so far. It is of great importance to conduct such research in a prospective manner, in order to increase quality and minimise bias. As such studies increase and achieve a large number of total patients, specific guidelines for each condition may be more applicable. By now, such recommendations exist only for SLE and antiphospholipid syndrome. Until then, the management of such high-risk pregnancies remain mainly empirical.
